# Ecosystem engineering strengthens bottom-up and weakens top-down effects via trait-mediated indirect interactions

**DOI:** 10.1098/rspb.2017.0894

**Published:** 2017-09-20

**Authors:** Zhiwei Zhong, Xiaofei Li, Dean Pearson, Deli Wang, Dirk Sanders, Yu Zhu, Ling Wang

**Affiliations:** 1 Institute of Grassland Science/School of Environment, Northeast Normal University, and Key Laboratory of Vegetation Ecology/Key Laboratory for Wetland Ecology and Vegetation Restoration, Changchun, Jilin 130024, China; 2 Rocky Mountain Research Station, USDA Forest Service, Missoula, MT 59801, USA; 3 Division of Biological Sciences, University of Montana, Missoula, MT 59812, USA; 4 Environment and Sustainability Institute, College of Life and Environmental Sciences, University of Exeter, Penryn Campus, Penryn, Cornwall TR10 9FE, UK

**Keywords:** bottom-up effects, density-mediated indirect effects, ecosystem engineering, top-down effects, trait-mediated indirect effects

## Abstract

Trophic interactions and ecosystem engineering are ubiquitous and powerful forces structuring ecosystems, yet how these processes interact to shape natural systems is poorly understood. Moreover, trophic effects can be driven by both density- and trait-mediated interactions. Microcosm studies demonstrate that trait-mediated interactions may be as strong as density-mediated interactions, but the relative importance of these pathways at natural spatial and temporal scales is underexplored. Here, we integrate large-scale field experiments and microcosms to examine the effects of ecosystem engineering on trophic interactions while also exploring how ecological scale influences density- and trait-mediated interaction pathways. We demonstrate that (i) ecosystem engineering can shift the balance between top-down and bottom-up interactions, (ii) such effects can be driven by cryptic trait-mediated interactions, and (iii) the relative importance of density- versus trait-mediated interaction pathways can be scale dependent. Our findings reveal the complex interplay between ecosystem engineering, trophic interactions, and ecological scale in structuring natural systems.

## 1. Introduction

Ecosystem engineers are defined as ‘organisms that modulate the availability of resources to other species by causing physical state changes in biotic or abiotic materials’ [1,2]. By manipulating the environment, ecosystem engineers can have powerful influences over species abundance, distribution, and diversity [3–5]. Given the importance of engineering and the fact that almost all organisms modify their environment to some extent [4], determining how ecosystem engineering affects other species, and the direction and magnitude of these effects, is critical for fully understanding community structuring. Despite the importance of ecosystem engineering as a structuring force, it has not been well integrated into community and food-web ecology [6–8]. Hence, we know remarkably little about how these powerful interactions structure natural communities.

One fundamental set of community interactions substantially influenced by engineering is trophic interactions [7–9]. By modifying habitats, ecosystem engineers can alter trophic interactions via two primary pathways. First, engineering can alter the abundances of organisms to influence trophic outcomes via density-mediated interactions or interaction chains [10–14]. Second, engineering can modify trophic interactions by changing behaviours or other traits of organisms that affect the *per capita* interaction strength between organisms at different trophic levels, thereby driving trait-mediated interactions [15–18]. These two pathways are not mutually exclusive and generally occur simultaneously in real ecosystems.

An important focus of indirect effects research has been to try to understand the relative contributions of density- versus trait-mediated pathways in driving top-down effects [18,19]. Whereas historically it was assumed that density-mediated effects were primary drivers of indirect effects, more recent studies partitioning density and trait effects suggest that trait-mediated effects can be as strong as or stronger than density effects [18]. However, an important consideration regarding these conclusions is the fact that this body of research is based primarily on microcosm experiments [20–22], which constrain the abundances of organisms in space and time and do not allow for scale-dependent interactions between density and trait effects. Fully understanding the interplay between trait- and density-driven processes requires experiments that examine trait and density pathways while allowing all factors to vary over more realistic spatial and temporal scales [23].

Large herbivores present one of the most influential groups of ecosystem engineers in terrestrial ecosystems [24,25]. These animals frequently alter plant community structure [26,27] with substantial effects on trophic interactions [28–30]. An important set of trophic linkages susceptible to the influences of large herbivore activities are predator–herbivore–primary producer interactions involving arthropod assemblages and herbaceous plants. For many arthropods, vegetation structure plays a vital role in regulating both their population dynamics and behavioural traits. For example, vegetation structure may influence the abundance and/or web size of web-building spiders by providing attachment points [31,32], which can in turn alter the strength of their top-down effects via both density- and trait-mediated pathways [33,34]. Notably, changes in vegetation structure may arise from changes in plant morphologies within species or from compositional changes that shift plant abundances between species of differing morphologies [31–34]. Similarly, vegetation structure may affect the abilities of invertebrate predators and herbivores to encounter their respective prey and host plants [35,36]. In addition to vegetation properties, grazing-induced alterations in microclimatic conditions could also influence arthropod abundances and their interactions [37]. Large herbivores and arthropods are widespread and play important roles in structuring natural and managed systems around the globe, particularly grassland and savannah ecosystems [38–40]. Hence, elucidating the effects of large herbivore engineers on invertebrate trophic interactions is essential to fully understand the structuring of these systems.

Here, we examined how a herbivore ecosystem engineer can indirectly alter trophic interactions in an arthropod-grassland food web. The system studied was comprised of a widespread herbivore ecosystem engineer, domestic sheep (*Ovis aries*), a predaceous web-building spider, *Argiope bruennichi*, its herbivorous grasshopper prey, *Euchorthippus* spp., and the grasshopper's host plant, *Leymus chinensis*. The aim of this study was to examine how engineering effects of the large herbivores influenced trophic interactions between spiders, grasshoppers, and grasshopper host plants while evaluating both density- and trait-mediated interaction pathways. To achieve this goal, we first executed a large-scale grazing experiment to examine how domestic sheep engineering affected the structure of grassland plant communities and the overall abundance of the web spiders and their grasshopper prey. We then used results from the large-scale study to develop small-scale microcosm experiments to explore plausible mechanisms underlying engineering effects on interactions between spiders, grasshoppers, and grasshopper host plants.

## 2. Material and methods

### (a) Study system and background

Our study was conducted at the Grassland Ecological Research Station of Northeast Normal University, located in a semi-arid low elevation grassland in Jilin Province, northern China (44°45′ N, 123°45′ E). The study site is dominated by the perennial grass *Leymus chinensis*, which accounts for 50–70% of the total aboveground vegetation biomass [41]. The dominant forbs are *Artemisia* (*A. scoparia*, *A. mongolica*, and *A. anethifolia*), which grow interspersed with *L. chinensis* in this grassland. Other common species include the grasses *Phragmites australis*, *Calamagrostis epigejos*, and *Chloris virgata*; legumes *Lespedeza davurica* and *Medicago ruthenica*; and forbs *Kalimeris integrifolia* and *Potentilla flagellaris*.

Domestic sheep are the dominant large herbivores in the study area. They prefer forb species and rarely feed on *L. chinensis* [42]. The major invertebrate herbivores are grasshoppers (Orthoptera, Acrididae) and planthoppers (Homoptera, Cicadellidae). Two grasshopper species, *Euchorthippus cheui* and *E. unicolor*, dominate the insect herbivore community, annually accounting for more than 65% of all insects. *E. cheui* and *E. unicolor* appear in late June and reach peak adult densities in mid-August. These species have similar body sizes and both feed predominately on *L. chinensis*. Since *E. cheui* and *E. unicolor* are ecologically similar and difficult to distinguish in the field, we treated them as a species complex (henceforth ‘*Euchorthippus*’). Key predators of *Euchorthippus* are spiders, birds, and robber flies. The orb-weaver *Argiope bruennichi* (Araneae: Araneidae) is a dominant spider species in our system which preys upon *Euchorthippus*. This species appears from June to October, attaining peak density (0.4–2.0 individuals m^−2^) in mid-August. Female *A. bruennichi* make vertical spiral orb webs usually among *L. chinensis* plants. These spiders capture and consume a variety of taxa, including grasshoppers. As with most web spiders, adult male *A. bruennichi* rarely build webs as they primarily seek mates.

Our experimental design integrated large-scale field experiments with complementary microcosm experiments. The large-scale experiments manipulated and quantified the overall effects of sheep grazing on abiotic conditions, plant community structure, *Euchorthippus* abundance, and *A. bruennichi* abundance and behaviours. The microcosm experiments controlled for target organism abundances to examine behavioural interactions between *A. bruennichi*, *Euchorthippus*, and *L. chinensis* in response to simulated forb removal, because large-scale experiments suggested that behavioural responses to forb removal via grazing was the mechanism underlying engineering effects on trophic interactions. The large-scale sheep grazing treatments were initiated early in May 2010 and consisted of six 20 × 30 m fenced exclosures that precluded sheep grazing (control treatment) paired with six 20 × 30 m unfenced plots with sheep access (grazed treatment) randomly located across the study area at 50–250 m intervals (electronic supplementary material, figure S1). From 2010 through 2012 (3 years), the study area was seasonally grazed by sheep from June through September at stocking rates of 0.1–0.3 animal units ha^−1^. Starting in 2013, large herbivores were excluded from the entire study area for grassland management objectives. In August 2014, after a period of sheep absence, we quantified the engineering effects from sheep grazing on plants, grasshoppers, and spiders in the 20 × 30 m control and grazed plots. System responses were quantified after sheep were removed to isolate engineering effects from the physical presence of sheep. However, we also quantified grazing effects on plant communities in August 2012, the last year of grazing, to establish the linkage between the grazing treatment and the post-grazing engineering effects.

Microcosm experiments were also conducted in August 2014. Microcosm experiments were created using large cages (1.2 m high × 2 m^2^ bottom surface area, covered with a 5 × 5 mm plastic mesh window screen) that enclosed preset densities of spiders and grasshoppers. One cage was randomly located within each control and sheep-grazed plot, totalling 6 control and 6 treatment cages, to evaluate behavioural responses to the large-scale grazing treatments (electronic supplementary material, figure S1). In addition, within each control plot, where vegetation had not been affected by sheep grazing, a similar cage was added within which forbs had been removed by hand clipping to examine the effects of forb removal on behavioural interactions relative to the un-manipulated control cage.

### (b) Quantifying effects of sheep grazing on plant community structure and microclimates in large-scale experiments

From August 12 to 17, 2014, we quantified microclimatic conditions and the structural attributes of key plant groups across the large-scale grazing treatments. We established two parallel transects (26 m long and 4 m apart) within each of the control and grazed plots and assessed microclimatic conditions and plant community properties in eight 1 × 1 m quadrats located every 2 m along each transect. Within each quadrat, we measured plant cover, density, and height overall for *L. chinensis*, other grasses, and forbs. Plant cover was visually estimated as the percentage of ground surface covered by each plant group within each quadrat. Plant density was estimated by counting the number of stems of each plant group within each quadrat. Plant height (cm) was measured on five haphazardly chosen stems for each plant group within each quadrat. Along the same transects, we assessed microclimate conditions by measuring air temperature and relative humidity at the ground surface and 30 cm above the surface within each quadrat from 10.00 to 16.00 hours, using an AR-847 digital thermo-hygrometer (Jinzhan Inc., Shenzhen, China). The average values of each plant community variable, and microclimate variable in the two transects were used for statistical analysis, providing a single data point for each variable in each 20 × 30 m plot.

### (c) Quantifying effects of sheep grazing on spiders and grasshoppers in large-scale experiments

In August 6 and 21, 2014 (sunny days), we measured the densities of *A. bruennichi* and *Euchorthippus* across the large-scale grazing treatments. We established two new parallel transects (26 m long and 4 m apart) 2 m to the side of the two vegetation survey transects within each plot to avoid the potential influences of vegetation surveys on arthropod density and behaviours. We randomly located 10 0.50 m^2^ rings (located every 1.5 m) along each transect and left the rings undisturbed for 3 days before sampling. We conducted the density surveys by slowly walking along each transect and counting the number of *A. bruennichi* webs and *Euchorthippus* within each ring. In the field, one *A. bruennichi* spider web is typically occupied by one *A. bruennichi* spider, so the number of webs and spiders is equivalent. In addition, we quantified *A. bruennichi* behaviours and predation successes by measuring the size (diameter in cm) and height (height from ground to web centre in cm) of each web and the number of *Euchorthippus* captures in each web. We calculated areas of the circular webs as *π* × radius^2^. We calculated *A. bruennichi* predation successes as: the total number of *Euchorthippus* captures in the spider webs we surveyed/the number of spider webs we surveyed in each transect. Quantifying *Euchorthippus* grasshopper behaviours in the open field was impractical so these were only quantified in microcosm cages as described below. We averaged the data for each transect from the two survey periods and then averaged those results between the two transects, providing a single data point in each 20 × 30 m plot for analyses for each variable.

### (d) Quantifying effects of sheep grazing on behavioural interactions in microcosms

Microcosm experiments were initiated 26 August 2014, five days after the field surveys of the large-scale grazing experiments. First, we assigned cage locations and quantified the same microclimatic and vegetation variables as in the large-scale plots using the methods described above. Next, the cage locations were cleared of invertebrates using a Univac Portable Suction Sampler (Burkard Co. Ltd, Rickmansworth, Herts, UK). Once cleared, the cages were set in place and the targeted species released into the cages. First, we stocked 40 late-instar *Euchorthippus* nymphs (20 males and 20 females) into each cage. This number was approximately 1.3 times their average densities on the control and grazed plots at the beginning of this experiment. We stocked nymphs rather than adults because most *Euchorthippus* in the study areas at this time were late-instar nymphs. Two days after stocking the grasshoppers, we introduced two adult female *A. bruennichi* into each cage. All organisms were collected from near the study area. We ran the experiment for 10 days.

Two days after initial stocking, we quantified web-building behaviours of *A. bruennichi* by measuring web area and height as described above. Additionally, we quantified interaction strength between the predator and prey by measuring *per capita* predation rates of *A. bruennichi* on *Euchorthippus*. Each day, we examined all 18 cages and recorded how many grasshoppers were captured by the spiders (webs). Within each cage, *A. bruennichi*'s predation rates on grasshoppers was calculated as the total number of grasshoppers captured per spider (web) during the 10-day experiment. For *Euchorthippus*, we quantified behaviours that might affect their interactions with their predators and their host plants. The behaviours were: (i) feeding: grasshopper observed eating foliage without retracting the head from the plants, (ii) walking: grasshopper observed walking on plants or the ground, and (iii) jumping: grasshopper observed jumping over the ground or among plants. During the 10-day experiment, we randomly selected one grasshopper from each cage for 1 day, and observed and recorded the frequency of each behaviour from 07.00–09.00, 11.00–13.00, and 15.00–17.00 hours (total 6 h/day). Before the beginning of observations, the selected grasshopper was given an identifying pin paint mark on its thorax. The paint mark allowed observers to see and relocate the targeted grasshopper in the green plant communities. During the observation period, the observer sat close to the focal cage, and monitored grasshopper behaviours from the observation window on the side of the cage.

### (e) Statistical analyses

Statistical analyses were performed in the open source software R 3.1.0 [43]. For the large-scale experiments, we used linear mixed effect models (lme) with grazing treatment treated as a fixed effect and replicate sites as a random effect to assess the impact of sheep grazing on plant cover, density, and height for each plant group and across all groups combined (*L. chinensis*, other grasses, forbs), air temperature and relative humidity (at ground surface and 30 cm above), densities of *A. bruennichi* and *Euchorthippus*, *A. bruennichi* web area and height, and *A. bruennichi* predation successes (mean number of grasshoppers caught per spider web). These were done using the function lme from the package nlme. For the microcosm experiments, we used similar linear mixed effects models with cage treatment treated as fixed factors and the replicates as random factors to test for effects of sheep grazing and forb removal effects on the same plant, abiotic, web variables, as well as *Euchorthippus* feeding, walking, and jumping frequencies. *A. bruennichi* predation rates (proportion of grasshoppers caught by spiders) were analysed with general linear mixed effects models (glme) including treatment as a fixed factor and replicate sites as a random factor assuming binomial error structure. The response variable was a binary variable containing (i) the number of grasshoppers caught in the spider webs and (ii) the number of grasshoppers that were not caught. Tukey tests for between treatment comparisons were performed with the function lsmeans from the lsmeans package for the microcosm experiments. All response variables were tested for normality and homogeneity of variance and log or square root transformed if necessary. Response variables with unequal variances for treatment groups were analysed using linear models based on generalized least squares (lmgls) where errors are allowed to have unequal variances, provided by the nlme package. We used VarIdent to account for variance heterogeneity in effect sizes between treatment groups.

## 3. Results

### (a) Effects of sheep grazing on plant community structure and microclimates in large-scale experiments

**Figure 1.**
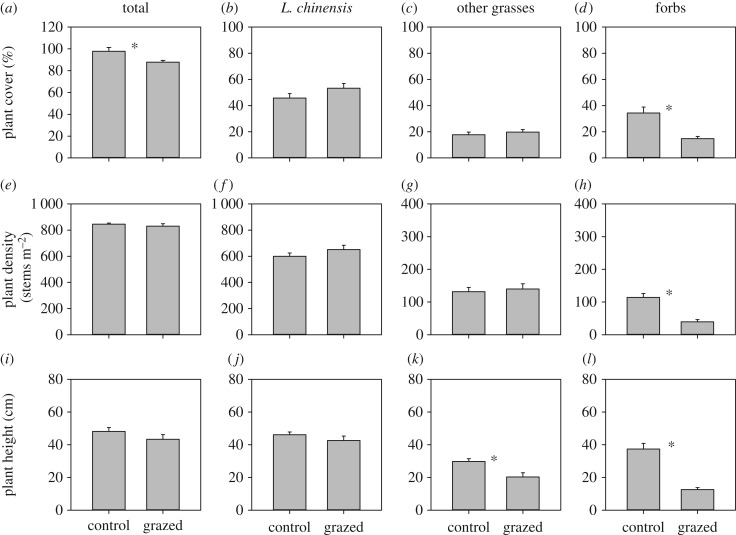
Effects of sheep grazing (control versus grazed plots) on plant community structure in large-scale experimental plots in August 2014 showing: (*a–d*) plant cover, (*e–h*) plant densities, and (*i–l*) plant heights for all plants combined (Total) and the three plant groups (*L. chinensis*, other grasses, and forbs). An asterisk (*) indicates significant differences between treatments. Error bars represent ±1 s.e.

Sheep grazing significantly decreased the cover (lme, *F*_1,5_ = 27.19, *p* = 0.003), density (lme, *F*_1,5_ = 50.91, *p* < 0.001), and height (lme, *F*_1,5_ = 61.27, *p* < 0.001) of forbs by 56%, 65%, and 66% (figure 1*d*,*h*,*l*), respectively. Sheep grazing significantly decreased total plant cover (lmgls, *F*_1,5_ = 6.17, *p* = 0.032; figure 1*a*), but it did not affect total plant density (lmgls, *F*_1,5_ = 0.56, *p* = 0.472) or mean plant height (*F*_1,5_ = 1.74, *p* = 0.245) in the plots (figure 1*e*,*i*). Grazing tended to increase the cover and density of the *L. chinensis* grass, but these effects were not significant (figure 1*b*,*f*). Grazing did not affect *L. chinensis* height (lmgls, *F*_1,5_ = 1.23, *p* = 0.296; figure 1*j*), but significantly decreased the height of other grasses by 32% (lmgls, *F*_1,5_ = 9.48, *p* = 0.012; figure 1*k*). Grazing did not affect air temperature or relative humidity at both ground surface and 30 cm above the ground in the plots (electronic supplementary material, figure S3*a*,*b*).

### (b) Effects of sheep grazing on spiders and grasshoppers in large-scale experiments

**Figure 2.**
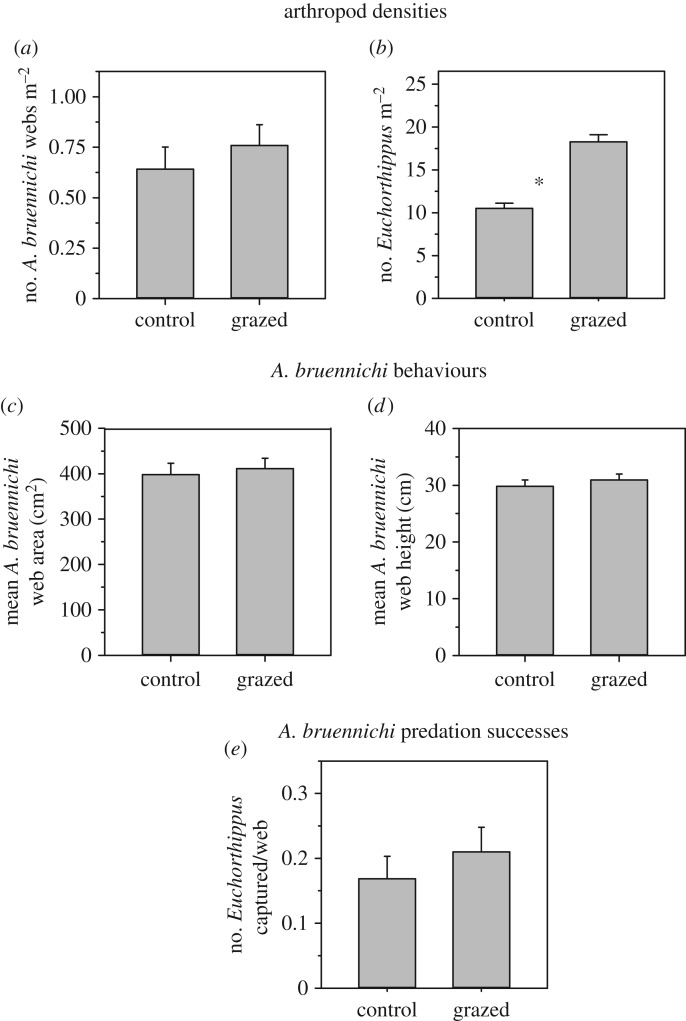
Effects of sheep grazing on (*a*) *A. bruennichi* spider density and web density (web and spider densities are equivalent), (*b*) *Euchorthippus* grasshopper density, (*c*) mean area and (*d*) mean height of *A. bruennichi* webs, and (*e*) predation successes of *A. bruennichi* on *Euchorthippus* in large-scale control and grazed plots in August 2014. An asterisk (*) indicates significant differences between treatments. Error bars represent ±1 s.e.

Sheep grazing did not alter the density of *A. bruennichi* (lme, *F*_1,5_ = 0.60, *p* = 0.473; figure 2*a*), however, it nearly doubled *Euchorthippus* densities in the grazed compared to the control plots (lme, *F*_1,5_ = 57.98, *p* < 0.001; figure 2*b*). Grazing did not alter *A. bruennichi* behaviour as measured by web size (*F*_1,5_ = 0.15, *p* = 0.714; figure 2*c*) or web height (*F*_1,5_ = 0.53, *p* = 0.500; figure 2*d*) or predation successes on *Euchorthippus* prey (*F*_1,5_ = 0.66, *p* = 0.455; figure 2*e*) in the plots.

### (c) Effects of sheep grazing on behavioural interactions in microcosms

**Figure 3.**
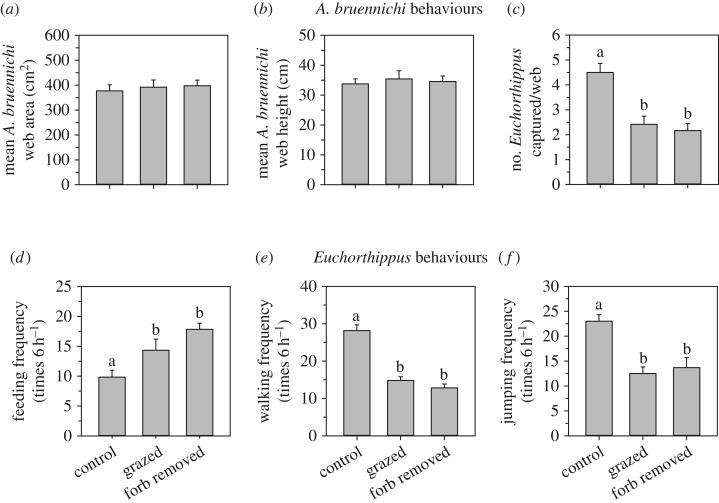
Effects of sheep grazing and forb removal on *A. bruennichi* spider and *Euchorthippus* grasshopper behaviours and interactions in microcosm experiments in August 2014 showing: *A. bruennichi* (*a*) mean web area, (*b*) mean web height, and (*c*) *per capita* predation rates on *Euchorthippus*; and *Euchorthippus* (*d*) feeding, (*e*) walking, and (*f*) jumping frequencies. Different letters above the bars indicate significant differences among treatments. Error bars represent ±1 s.e.

The patterns of plant community structure and microclimates in the microcosm experiments were similar to those measured in the large-scale grazing experiments (electronic supplementary material, figures S2 and S3). As with the large-scale experimental results, grazing and forb removal did not affect *A. bruennichi* behaviours based on spider web area (lme, *F*_2,10_ = 0.17, *p* = 0.844; figure 3*a*) and web height (lme, *F*_2,10_ = 0.16, *p* = 0.851; figure 3*b*) in the microcosm experiments. However, we found significant treatment effects on predation rates of *A. bruennichi* (lme, *F*_2,10_ = 16.49, *p* = 0.001). Predation rates by *A. bruennichi* on *Euchorthippus* decreased by 46% in the grazed versus the control treatment (glme, *z* = −2.98, *p* = 0.003; figure 3*c*), and this pattern was paralleled by a 52% decrease in predation rates in the forb removal versus the control treatment (glme, *z* = −3.365, *p* < 0.001; figure 3*c*). Sheep grazing and forb removal treatments substantially altered *Euchorthippus* behaviours based on feeding frequency (lme, *F*_2,10_ = 8.18, *p* = 0.008), walking frequency (lme, *F*_2,10_ = 47.92, *p* < 0.001), and jumping frequency (lmgls, *F*_2,10_ = 17.41, *p* < 0.001). *Euchorthippus* feeding frequency in the grazed treatment increased by 46% compared to the control treatment (lme, *t*_1,10_ = 2.270, *p* = 0.047), and *Euchorthippus* feeding frequency in forb removal treatment increased by 81% compared to the control treatment (lme, *t*_1,10_ = 4.03, *p* = 0.002; figure 3*d*). Sheep grazing and forb removal lowered the walking frequency of *Euchorthippus* by 47% and 54%, respectively (lme, *t*_1,10_ = −7.83, *p* < 0.001; *t*_1,10_ = −9.00, *p* < 0.001; figure 3*e*), and jumping frequency by 46% and 41%, respectively (lmgls, *t*_1,10_ = −5.60, *p* = 0.0001; *t*_1,10_ = −3.86, *p* *=* 0.0015; figure 3*f*).

## 4. Discussion

Ecosystem engineering is a ubiquitous and powerful phenomenon which influences a wide range of ecological interactions [2,4,8]. Yet, the mechanisms by which engineering affects food web structure and trophic interactions are poorly understood. By integrating large-scale field experiments with microcosm experiments, we were able to identify prospective mechanisms underlying a large herbivore's ecosystem engineering effects and elucidate the indirect effect pathways by which engineering shifted trophic interactions in a grassland food web. Our results suggest that selective sheep grazing generated engineering effects that strengthened bottom-up and weakened top-down interactions by altering cryptic trait-mediated interactions between grasshoppers and their host plants and between grasshoppers and their spider predators. We also found that changes in grasshopper behaviours which reduced spider *per capita* predation in the microcosms did not translate to reduced spider predation successes at the larger system scale, suggesting that increased grasshopper densities at larger spatial and temporal scales offset reductions in risky individual grasshopper behaviours. Our results provide important insights regarding how engineering can drive trait-mediated indirect interactions by altering the arenas for predator–prey and plant–herbivore interactions and how ecological scale can influence the relative importance of trait- versus density-mediated processes.

### (a) Engineering effects on the overall community interaction web

**Figure 4.**
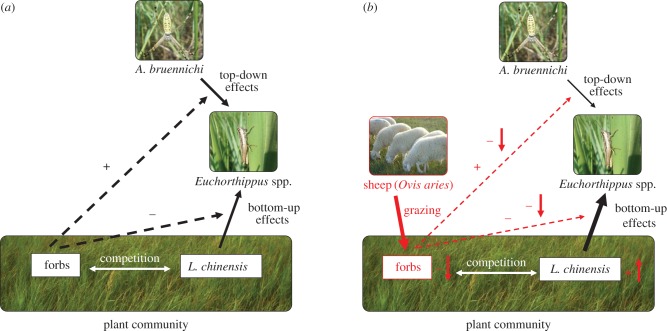
Community interaction web showing the effects of ecosystem engineering from sheep grazing on east-Asian steppe communities. In the absence of sheep (panel *a*), forbs strongly influence interactions (interaction modifications, indicated as black dashed lines) (i) between *Euchorthippus* grasshoppers and their host *L. chinensis* grass by interfering with grasshopper foraging and weakening plant–herbivore interactions (indicated as black ‘−’), and (ii) between grasshoppers and their *A. bruennichi* spider predators by facilitating grasshopper walking and jumping behaviours that drive grasshoppers into spider webs and strengthen predator–prey interactions (indicated as black ‘+’). In the presence of sheep (panel *b* with sheep effects indicated in red), selective grazing of sheep strongly reduces forb abundance and height (indicated as ‘−↓’ next to the forb group) which alters the arena for these interactions, weakening the forb's facilitative effects on predator–prey interactions and its interference effects on plant–herbivore interactions (indicated as ‘−↓’ applied to the two red dashed lines). The overall effect of ecosystem engineering by sheep is an increase in strength of bottom-up (indicated as a thicker solid line in black between *L. chinensis* and *Euchorthippus*) and decrease in strength of top-down effects (indicated as a thinner solid line in black between *A. bruennichi* and *Euchorthippus*) mediated by interaction modifications.

The effects of engineering in this system were revealed by the complementarity of the large- and small-scale experiments aided by the fact that key aspects of the system are naturally compartmentalized. The large-scale herbivore manipulation demonstrated that grazing had two primary effects. It directly reduced forb abundance/height and indirectly increased grasshopper abundance. The finding that grazing had minimal effects on plants other than forbs and no measurable effect on abiotic conditions, indicated that engineering effects were transmitted through reductions in forb abundance/height. The fact that the abundances of *Euchorthippus*'s host plant and its spider predator did not change, suggested that increases in *Euchorthippus* abundance were not caused by overt density-mediated indirect interactions. Microcosm experiments allowed us to manipulate forb abundance/height independent of grazing effects to mechanistically evaluate how changes in forb abundance/height might affect trait-mediated indirect interactions. Experimental removal of forbs resulted in (i) increased feeding by *Euchorthippus* on its host plant, (ii) reduced walking and jumping by *Euchorthippus*, (iii) fewer *Euchorthippus* captured in *A. bruennichi* webs, and (iv) no changes in *A. bruennichi* web construction, indicating no change in *A. bruennichi* hunting behaviours. These results suggest that forbs inhibited *Euchorthippus* foraging on their host plants and facilitated *A. bruennichi* predation on *Euchorthippus*, and that changes in these interactions were driven solely by changes in *Euchorthippus* behaviours. In sum, sheep grazing generated engineering effects via selective foraging on strongly interacting forbs which increased *Euchorthippus* abundance by modifying trait-mediated indirect interactions in ways that strengthened bottom-up and weakened top-down effects (figure 4).

### (b) Engineering effects on predator abundance and behaviour

Identifying the relative strength of density versus behavioural responses to engineering effects at each trophic level further highlights the mechanisms and processes underlying the community-level outcomes. *A. bruennichi* spiders did not exhibit density or behavioural responses (spider densities and web size were similar between grazing treatments; figures 2 and 3) to engineering despite changes in vegetation architecture and an increase in prey abundance. This finding was unexpected because spiders that use plants for web substrates often change in abundance and may alter web construction behaviours in response to vegetation changes [31–34], and spiders are often prey limited [44]. The lack of *A. bruennichi* response to vegetation changes was attributable to the fact that grazing affected the abundance and height of forbs, but failed to affect the quantity or morphology of *A. bruennichi*'s primary web substrate, *L. chinensis* (figure 1*b*,*f*,*j*). The failure of *A. bruennichi* to increase in abundance in response to increased prey densities could mean these spiders are limited by substrate availability more than food availability [33]. However, the fact that spider capture rates did not increase despite higher *Euchorthippus* prey densities, suggests that the changes in *Euchorthippus* behaviour demonstrated in the microcosms which reduced their risk of capture in webs may have countered the effects of higher prey densities. Of course, we did not measure all species in this system and it is possible that changes in other factors such as *A. bruennichi*'s predators or alternative prey may have come into play.

### (c) Engineering effects on plant abundance and ‘behaviour’

At the primary producer level, there was also no significant change in abundance or behaviours (morphology) of *L. chinensis* (figure 1*b*,*f*,*j*), the primary host plant for *Euchorthippus*. Given the dominance of *L. chinensis* over *Artemisia* forbs in these grasslands, it is possible that *L. chinensis* is little affected by forb competition. However, it is also possible that over longer time periods than 3 years of grazing, *L. chinensis* might increase in abundance in response to forb reductions. An alternative explanation is that the near doubling of *Euchorthippus* densities (figure 2*b*) combined with a near doubling of their *per capita* feeding rates on *L. chinensis* (figure 3*d*) may have offset any release it might experience from reduced competition with forbs. In either case, the vegetation response appeared to be stable as the patterns shown in the last year of grazing (see electronic supplementary material, figure S4, vegetation in 2012), were similar to those shown 2 years after grazing had ended (figure 1, vegetation in 2014).

### (d) Engineering effects on herbivore abundance and behaviour

The herbivore *Euchorthippus* showed the greatest sensitivity to engineering effects in this system, both in terms of density and behavioural responses (figures 2 and 3). This species doubled in population size in the large-scale experiment. Interestingly, the microcosm experiments suggested that these density responses were behaviourally driven. In microcosm experiments *Euchorthippus* substantially shifted its activity patterns in response to forb removal by reducing walking and jumping behaviours by 50% while nearly doubling its time spent feeding on *L. chinensis* (figure 3*d–f*). These results suggest that forb removal allowed *Euchorthippus* to increase its feeding time because it spent less time circumnavigating non-food plants to reach its host plant. Such interference by non-host plants in herbivore–host interactions is a common and potent source of defrayed herbivory [35,36]. This increased feeding likely contributed to the increased *Euchorthippus* densities observed in the large-scale sheep-grazing treatments (figure 2*b*). Meanwhile, reduced walking and particularly jumping behaviours were linked to a near 50% reduction in *Euchorthippus* captures in *A. bruennichi* webs (figure 3*c*). This effect is attributable to the fact that webs are passive prey capture devices [33,34], hence reducing prey movement reduces the likelihood of encountering and becoming ensnared in a web. This reduction in predation risk may have also contributed to the observed increase in *Euchorthippus* densities at the system level*.* Overall, results from the microcosm experiments suggested that the increase in *Euchorthippus* densities observed in the large-scale experiments resulted from engineering effects strengthening bottom-up effects and weakening top-down effects via changes in *Euchorthippus* behaviour.

### (e) Ecological scale and the interplay between density- and trait-mediated interactions

Our approach of integrating large- and small-scale field experiments generated novel insights regarding the role of ecological scale on the interplay between density- and trait-mediated interactions. Our microcosm experiments indicated that engineering reduced the susceptibility of individual *Euchorthippus* to *A. bruennichi* predation by nearly 50% (figure 3*c*). Yet, this effect was not reflected in the large-scale field experiments (figure 2*e*), where spider predation on *Euchorthippus* did not differ between the grazed and control treatments. One simple explanation for this pattern could be that the near 50% reduction in grasshopper predation rates resulting from reductions in risky individual grasshopper behaviours was offset by a doubling of grasshopper densities arising from increased foraging activity accumulating over more natural timescales. Our microcosm experiments were not sophisticated enough to tease out exactly how these changes in *Euchorthippus* behaviours interacted with changes in their densities because we did not manipulate *Euchorthippus* densities. Nonetheless, the combination of the two experimental approaches suggests a complex interplay between trait and density effects wherein trait-mediated interactions may alter species' densities over longer time frames and/or larger spatial scales in ways that influence how trait and density effects play out at the system level. Most inferences regarding the relative roles of density- versus trait-mediated interactions have been derived from small-scale, short-term, microcosm-type experiments [19,21,22,45]. Such studies tend to control densities of organisms in an effort to isolate density versus trait effects. However, density effects may dynamically interact with trait effects at larger system scales. Our results suggest that fully understanding the relative role of density- and trait-mediated effects in ecological systems will require better linking microcosm experiments to the systems they are intended to reflect.

## Supplementary Material

Fig. S1 The experimental design.; Fig. S2 Vegetation in the small-scale experiments in August 2014.; Fig. S3 Microclimates in the both large- and small-scale experiments in August 2014.; Fig. S4 Vegetation in the large-scale experiments in August 2012.

## Data accessibility

The data used for this study are available from the Dryad Digital Repository at http://dx.doi.org/10.5061/dryad.7v475 [46].
