# Supplementary material for: Ecosystem engineering strengthens bottom-up and weakens top-down effects via trait-mediated indirect interactions
Source: Proc Biol Sci. 2017 Sep 20;284(1863):20170894. doi: 10.1098/rspb.2017.0894 (PMC5627195; doi:10.1098/rspb.2017.0894)
Supplement: Fig. S1 The experimental design.; Fig. S2 Vegetation in the small-scale experiments in August 2014.; Fig. S3 Microclimates in the both large- and small-scale experiments in August 2014.; Fig. S4 Vegetation in the large-scale experiments in August 2012. [file rspb20170894supp1.doc]

**supplementary materials**

**Ecosystem engineering strengthens bottom-up and weakens top-down effects via trait-mediated indirect interactions**

**Zhiwei Zhong^1^, Xiaofei Li ^1^, Dean Pearson^2,3^, Deli Wang^1^, Dirk Sanders^4^, Yu Zhu^1^, Ling Wang^1^**

*^1^Institute of Grassland Science/School of Environment, Northeast Normal University, and Key Laboratory of Vegetation Ecology, Ministry of Education, Changchun, Jilin 130024, China*

*^2^Rocky Mountain Research Station, USDA Forest Service, Missoula, MT 59801, USA*

*^3^Division of Biological Sciences, University of Montana, Missoula, MT 59812, USA*

*^4^Environment and Sustainability Institute, College of Life and Environmental Sciences, University of Exeter, Penryn Campus, Penryn, Cornwall, TR10 9FE, UK*

**Journal name:** Proceedings of the Royal Society B: Biological Sciences

**Article DOI:** 10.1098/rspb.2017.0894.

**FIGURE S1**


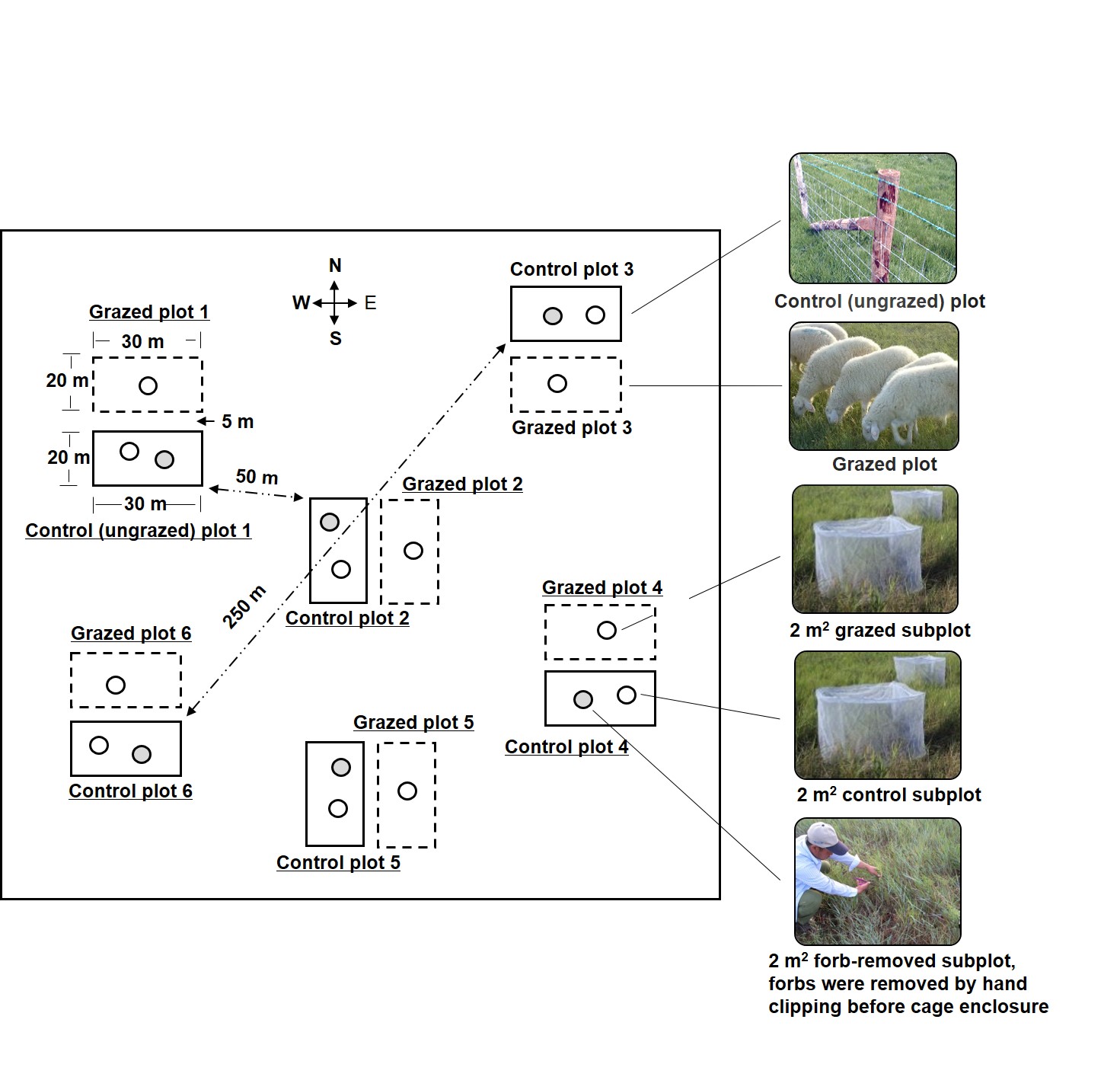


FIGURE S1 The experimental design for this study in a semi-arid low elevation grassland in Jilin Province in northern China. There are six 20 × 30 m large-herbivore exclosures (control plots) separated by 50-250 m located randomly across the study site. These control plots were established before the beginning of grazing activities in 2010. From 2010 through 2012, the study area was seasonally grazed by sheep from June through September at stocking rates of 0.1 – 0.3 animal units/ha. In June 2014, one 20 × 30 m plot was randomly selected to serve as grazed plots in areas adjacent to each of the six control plots. In August 2014, we randomly designated one 2 m^2^ circular subplot into each of the control plots and each of the grazed plots. We also added a forb-removed treatment in the control plots to examine the potential effects of forbs on the behaviors of and trophic interactions between *A. bruennichi* and *Euchorthippus*, and between *Euchorthippus* and *L. chinensis* host grass, independent of sheep grazing in the field. As a result, this experiment consisted of three types of treatment subplots: control subplots, grazed subplots, and forb removed subplots. Each treatment replicated six times, resulting in a total of 18 subplots across the study site.

**FIGURE S2**


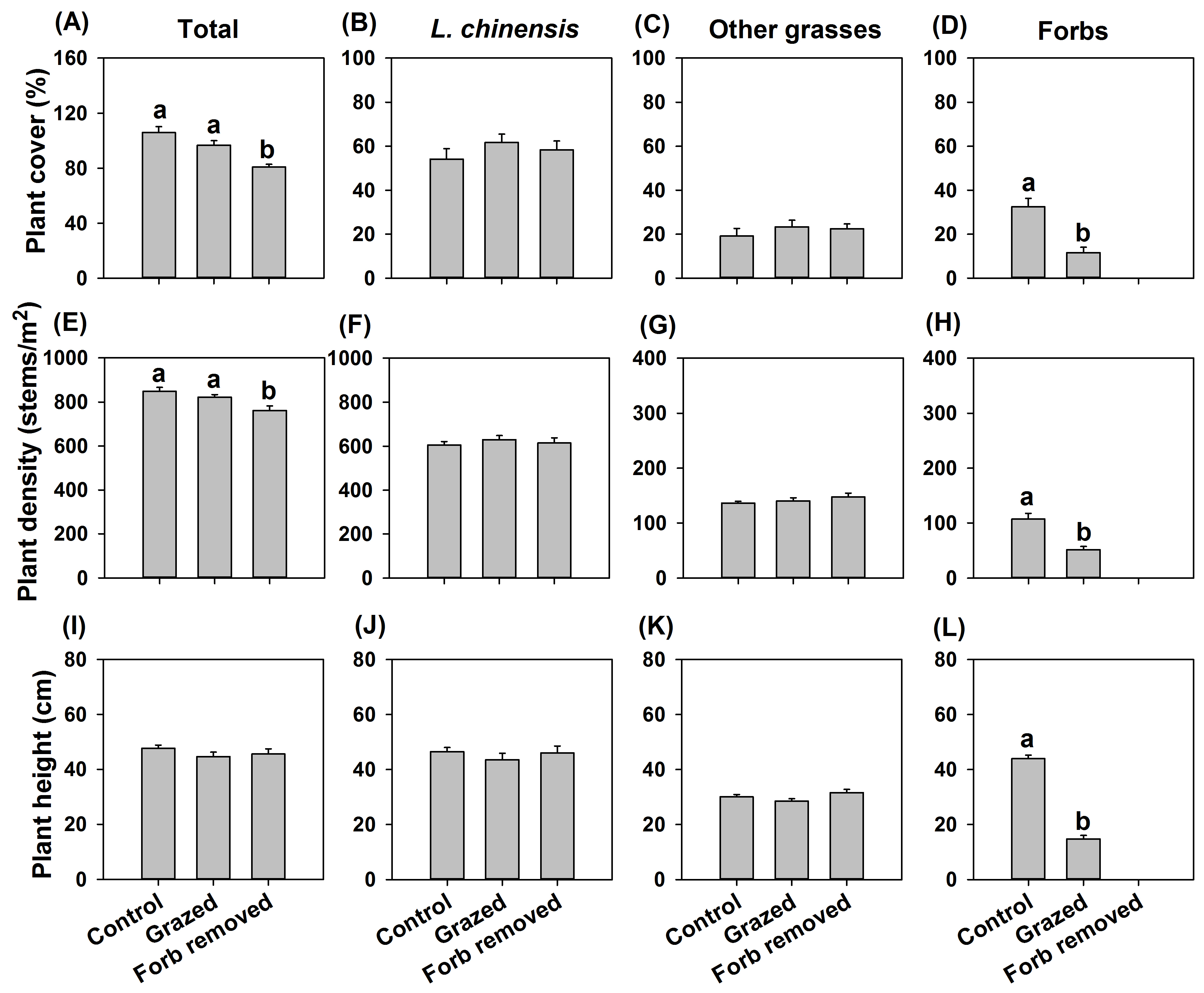


FIGURE S2 Effects of sheep grazing and forb removal on plant community structure in the microcosm experiments in August 2014: (A-D) plant cover, (E-H) plant densities, and (I-L) plant height of total and the three plant groups (*L. chinensis*, other grasses and forbs). Different letters above the bars indicate significant differences among treatments. Error bars represent ±1 SE.

Results for FIGURE S2: Linear mixed effects model (lme) analyses showed that sheep grazing prior to caging of the microcosm experiments caused significantly lower forb cover, forb density and forb height in the grazed treatment compared to the control treatment (forb cover, *F*_1,5_ = 25.00, *P* = 0.004; forb density, *F*_1,5_ = 21.44, *P* = 0.006; forb height, *F*_1,5_ = 342.71, *P* < 0.001; Fig. S2D, H, and L). In addition, we detected significant treatment effects on total plant cover (lme, *F*_2,10_ = 19.97, *P* < 0.001; Fig. S2A) and total plant density (lme, *F*_2,10_ = 12.61, *P* = 0.002; Fig. S2E). Forb removal significantly lowered total plant cover compared to the control treatment (Tukey test, *P* < 0.001), and the grazed treatment (Tukey test, *P* < 0.007; Fig. S2A). Similarly, forb removal significantly lowered total plant density compared to the control treatment (Tukey test, *P* = 0.002), and the grazed treatment (Tukey test, *P* = 0.018; Fig. S2E). We detected no treatment effects on mean plant height (Fig. S2I), plant cover, plant density or height of *L. chinensis* (Fig. S2B, F, J) and other grasses (Fig. S2C, G, K) in the microcosm experiments.

**FIGURE S3**


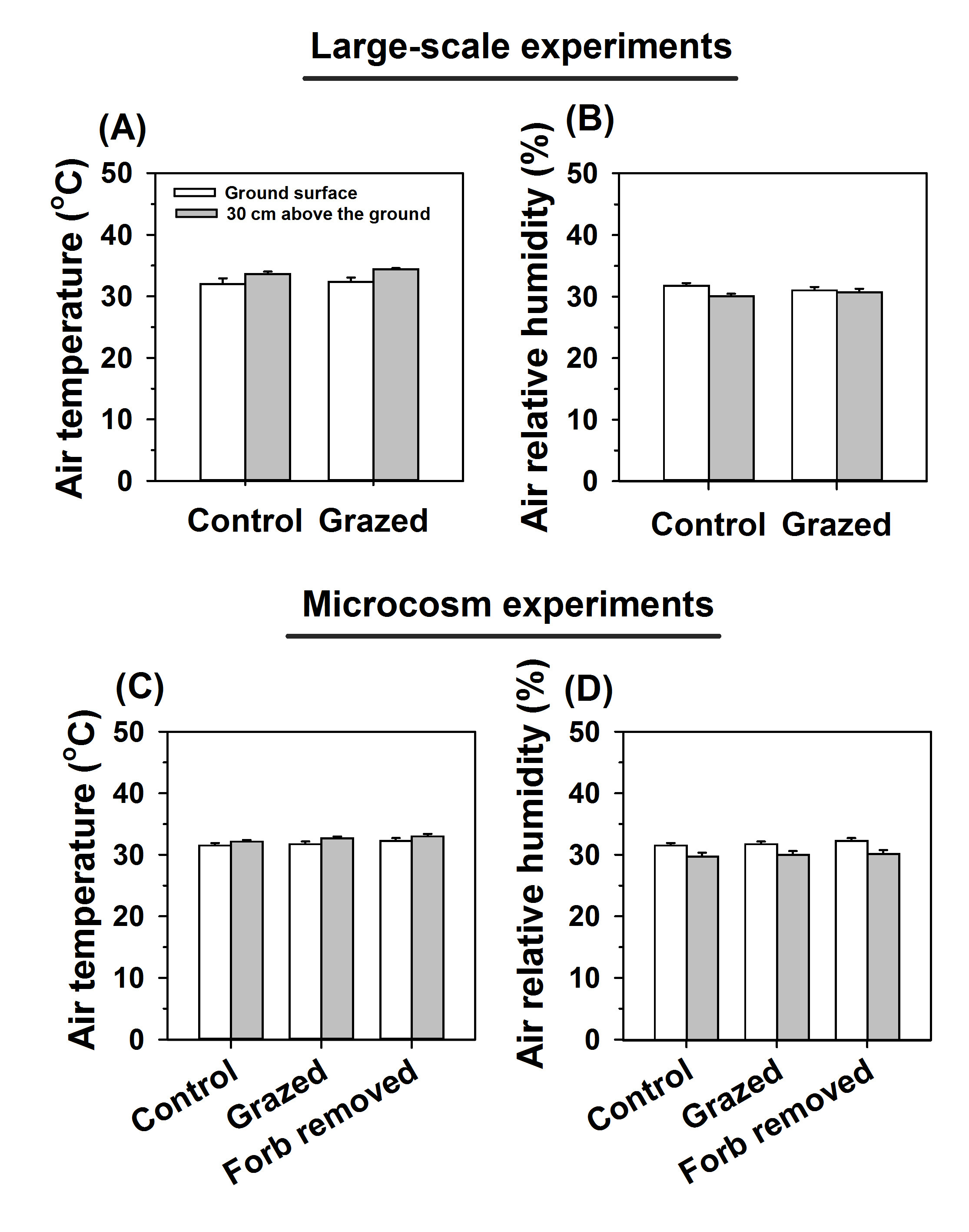


FIGURE S3 Effects of sheep grazing on the (A) air temperature, and (B) air relative humidity of ground surface and 30 cm above the ground in the large-scale experiments in August 2014, and effects of sheep grazing and forb removal on (C) air temperature, and (D) air relative humidity of ground surface and 30 cm above the ground in the microcosm experiments. Error bars represent ±1 SE.

Results for FIGURE S3: For the large-scale experiments, linear mixed effects model (lme) analyses showed that grazing did not affect air temperature (ground surface: lme, *F*_1,5_ = 0.77, *P* = 0.420; 30 cm above the ground: lmgls, *F*_1,5_ = 3.77, *P* = 0.081; Fig. S3A) or relative humidity (ground surface: lme, *F*_1,5_ = 0.53, *P* = 0.500; 30 cm above the ground: lme, *F*_1,5_ = 1.43, *P* = 0.287; Fig. S3B). For the microcosm experiments, lme analyses showed that grazing and forb removal did not affect air temperature (ground surface: *F*_2,10_ = 0.84, *P* = 0.461; 30 cm above the ground: *F*_2,10_ = 2.53, *P* = 0.129; Fig. S3C) and humidity (ground surface: *F*_2,10_ = 0.70, *P* = 0.522; 30 cm above the ground: *F*_2,10_ = 0.11, *P* = 0.896; Fig.S3D).

**Vegetation conditions in 2012**

Vegetation sampling in August 2012: During the peak of growing season (August) in 2012, the last year of sheep grazing treatment, we surveyed plant community structure in the grazed and ungrazed areas using the methods desribed in the main text. Please see the vegetation survey methods in the section of “*Quantifying effects of sheep grazing on plant community structure and microclimates in large-scale experiments*” in the main text.

Statistical analyses: Statistical analyses were performed in the open source software R 3.1.0 [43]. We used linear mixed effect models (lme) with grazing treatment treated as fixed effect and replicate sites as random effect to assess the impact of sheep grazing on plant cover, density, and height for each plant group and across all groups combined (*L. chinensis*, other grasses, forbs) in August 2012.

Results for effects sheep grazing on vegetation in 2012: Sheep grazing significantly decreased the cover (*F*_1,5_ = 45.06, *P* = 0.001), density (*F*_1,5_ = 94.23, *P* < 0.001), and height (*F*_1,5_ = 67.83, *P* < 0.001) of forbs by 80%, 78%, and 77% (Fig. S4D, H, L), respectively. Sheep grazing significantly decreased total plant cover (*F*_1,5_ = 11.17, *P* = 0.020; Fig. S4A), and mean plant height (*F*_1,5_ = 14.91, *P* = 0.012; Fig. S4E), but it did not affect total plant density in the plots (Fig. S4I). Grazing tended to increase the cover and density of the *L. chinensis* grass, but these effects were not significant (Fig. S4B, F). Grazing tended to decrease *L. chinensis* height, but this effect was not significant (*F*_1,5_ = 2.25, *P* = 0.194; Fig. S4J). Sheep grazing significantly decreased the height of other grasses by 16% (*F*_1,5_ = 13.65, *P* = 0.014; Fig. S4K).

**FIGURE. S4**

**
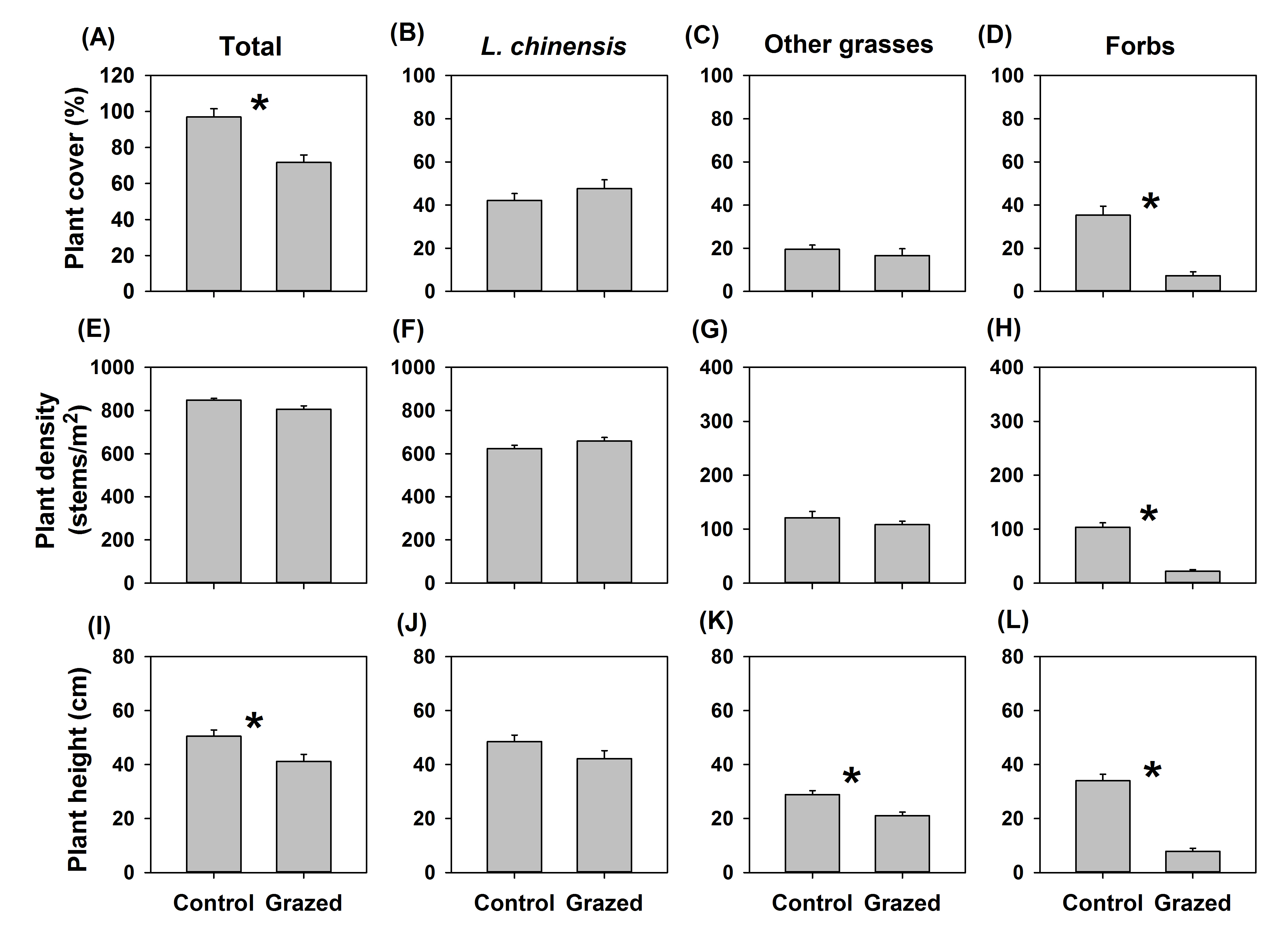
**

FIGURE S4 Effects of sheep grazing (control versus grazed plots) on plant community structure in large-scale experimental plots in August 2012 immediately following the sheep grazing period showing: (A–D) plant cover, (E–H) plant densities, and (I–L) plant heights for all plants combined (Total) and the three plant groups (*L. chinensis*, Other grasses and Forbs). An asterisk (*) indicates significant differences between treatments. Error bars represent ±1 SE.
